# Hippocampal microstructure as a measure of cognitive resilience to tau PET burden in older adults

**DOI:** 10.1016/j.tjpad.2025.100454

**Published:** 2026-01-06

**Authors:** Daniel D. Callow, Nisha Rani, Kylie H. Alm, Corinne Pettigrew, Michael Miller, Marilyn Albert, Arnold Bakker, Anja Soldan, the BIOCARD Research Team

**Affiliations:** a Department of Psychiatry and Behavioral Sciences, Johns Hopkins University School of Medicine, Baltimore, MD, USA; b Department of Neurology, Johns Hopkins School of Medicine, Baltimore, MD, USA; c Department of Biomedical Engineering, Johns Hopkins University, Baltimore, MD, USA

**Keywords:** Hippocampal mean diffusivity, Synaptic integrity, Neuroinflammation, Alzheimer’s disease, Braak stages

## Abstract

**Background:**

Cognitive resilience, the ability to maintain better than expected cognitive function despite neuropathological burden, is a key contributor to clinical outcomes in Alzheimer’s disease (AD), though the underlying neurobiological mechanisms remain poorly understood.

**Objectives:**

To determine whether hippocampal volume and microstructure moderate the relationship between early tau pathology and cognitive performance, thereby serving as potential markers of cognitive resilience.

**Design:**

Cross-sectional observational study.

**Setting:**

Participant data was obtained from the longitudinal BIOCARD Study, a volunteer-based research cohort.

**Participants:**

The sample included 190 dementia-free adults (mean age = 68 years), comprising 176 cognitively unimpaired individuals and 14 with mild cognitive impairment (MCI).

**Measurements:**

Hippocampal volume and microstructure (mean diffusivity (MD)) were measured using structural magnetic resonance imaging (MRI) and diffusion-weighted imaging (DWI), respectively. Tau pathology was measured using FMK-6240 tau PET imaging across Braak stages I–III. Cognitive performance was indexed using global and domain-specific composite scores. Regression models tested the interactions between hippocampal volume or MD and tau burden, adjusting for demographics, *APOE* genotype, amyloid status, and diagnostic status.

**Results:**

Lower hippocampal MD (indicative of better microstructural integrity) attenuated the negative association between tau burden in Braak stages II–III and both global cognition and episodic memory (*p*s < 0.010). Logistic regression models indicated that lower hippocampal MD was associated with a weaker relationship between tau burden in Braak stages II–III and the likelihood of MCI diagnosis (*p*s < 0.050). In contrast, hippocampal volume did not moderate the relationship between tau and any cognitive outcome (*p*s > 0.250).

**Conclusions:**

Hippocampal MD may serve as a promising imaging marker of cognitive resilience to early tau pathology, with potential utility for risk stratification and as a target for preventive interventions in AD.

## 1 Introduction

Alzheimer’s disease (AD) pathology, characterized by amyloid-β plaques and tau neurofibrillary tangles, is a primary driver of cognitive impairment [1,2]. However, there is considerable variability in the level of cognitive impairment among individuals with similar levels of AD pathological burden [3,4]. This phenomenon has given rise to the concept of cognitive resilience (or cognitive reserve) [4], a concept designed to explain why some individuals show better than expected cognitive performance given their degree of brain pathology or disease [5]. Although cognitive resilience is presumed to reflect underlying neurobiological processes [6,7], the specific mechanisms that confer this protection remain poorly understood. Clarifying these mechanisms is an important step in reducing clinical progression and cognitive decline, and for targeting interventions in individuals at higher risk for cognitive impairment.

Tau neurofibrillary tangles evaluated on autopsy typically begin accumulating in the transentorhinal cortex (Braak Stage I), progressing through the entorhinal cortex and into the hippocampus (Braak Stage II), before spreading to other regions of the medial temporal lobe and cortex (Braak stages III to VI) [8,9]. Tau tangle burden can also be measured in vivo using positron emission tomography (PET), with second-generation tracers like 18F-MK6240 offering high specificity for tau tangles and a broad dynamic range of standardized uptake value ratios (SUVRs), allowing for more precise quantification of tau burden in early Braak stages [10]. Although tau burden in early Braak stages is associated with poorer memory and cognitive impairment in older adults [11,12], the association between tau burden and cognitive performance or decline can vary substantially across individuals [13,14]. This suggests that additional factors, such as brain-based resilience mechanisms, may moderate the impact of tau pathology on cognition.

Several general brain mechanisms have been proposed to underly cognitive resilience to AD pathology, including having larger regional brain volumes and better microstructural integrity [15,16]. For instance, hippocampal volume has been cited as a marker of resilience [7,16], however, few studies have directly examined its moderating effect on the association between AD pathology and cognitive outcomes, leading to inconsistent findings [[16], [17], [18]]. Moreover, most prior work has focused on cerebrospinal fluid (CSF) and amyloid PET biomarkers, rather than tau PET pathology ^13^, which is more directly related to cognitive impairment [2,11,13], leaving a key gap in our understanding of resilience to tau-related neurodegeneration.

Recent investigations into microstructural properties have shown that better network-level white matter integrity, measured via diffusion weighted imaging (DWI), is associated with a reduced impact of tau PET burden on memory decline [19]. However, it remains unclear whether gray matter microstructural integrity similarly moderates the relationship between tau pathology and cognition. Notably, DWI-derived hippocampal measures, such as mean diffusivity (MD), are more strongly associated with memory performance and cognitive impairment compared to hippocampal volume [[20], [21], [22], [23]]. This suggests that DWI-based measures may be more sensitive indicators of cognitive resilience compared to volumetric measurements.

In line with the reserve and resilience framework[5], this study addresses existing gaps in our understanding of cognitive resilience by testing whether hippocampal volume and microstructure moderate the association between tau PET burden across Braak stages I–III and cognitive performance, specifically in global cognition, episodic memory, and visual-spatial processing, domains linked to hippocampal function [24]. We focused specifically on the hippocampus, as its gray matter microstructure can be accurately assessed with DWI [20,25] and has been linked to various lifestyle factors known to influence cognitive resilience [[25], [26], [27]]. Additionally, it is vulnerable to early tau accumulation associated with AD [8,9]. We hypothesized that lower hippocampal MD (indicating better microstructural integrity) would be associated with a weaker negative relationship between greater tau burden and cognitive performance. We also examined whether hippocampal volume and MD influence the association between tau burden and clinical status (i.e., cognitively unimpaired vs. Mild Cognitive Impairment (MCI)).

## 2 Methods

This analysis utilized data from the ongoing Biomarkers of Cognitive Decline among Normal Individuals (BIOCARD) study, a longitudinal cohort initiated in 1995 at the National Institutes of Health (NIH). The original cohort comprised 349 largely middle-aged adults, with approximately 75 % reporting a family history of dementia. During the first phase of the study, conducted at the intramural program of the NIH (1995–2005), clinical and cognitive assessments, CSF, blood, and 1.5 T MRI scans were acquired. After a pause from 2005 to 2009, the study resumed at Johns Hopkins University (JHU). Since then, participants have continued annual evaluations, including clinical assessments and cognitive testing. Neuroimaging, including 3T MRI (MPRAGE and DWI) has been conducted biennially since 2015. Amyloid PET scans began in 2015 and tau PET acquisition began in 2021. Recruitment of additional participants began in 2020 to increase the size and diversity of the cohort. Preliminary analyses showed that including an indicator variable for cohort (original (*n* = 94) vs. new enrollee (*n* = 96) did not change any results (data not shown) and therefore, the analyses presented below do not adjust for cohort.

This study is based on cross-sectional data from 190 participants without dementia (176 cognitively unimpaired and 14 with MCI), all of whom had amyloid and tau PET scans, DWI, and relevant cognitive data available. Cognitive data from the same visit as the tau PET scans were used when available (*n* = 185); otherwise, data collected within one year of the tau PET scan was included (*n* = 7). Additionally, MRI scans from the same visit as the tau PET scan were used when available (*n* = 156), otherwise the MRI scans collected closest in time to the tau PET scan was used (mean = 0.33 years; SD = 0.9 years, range = −1.3 - 4.9 years). Finally, amyloid and tau PET scans from the same visit were used, when possible (163), otherwise the amyloid scan collected closest in time to the tau PET scan was used (mean = 0.18 years; SD = 0.6 years, range = −1.3 – 2.9 years). Study activities were conducted in accordance with ethical standards of the responsible committee on human experimentation and with the Helsinki declaration of 1975, as revised in 2020.

### 2.1 Clinical and cognitive assessments

Participants undergo clinical evaluations and cognitive assessments at each annual visit, including a semi-structured interview based on the Clinical Dementia Rating (CDR) scale [28] and a battery of neuropsychological tests. A confirmatory factor analysis was performed based on 12 test scores obtained from the neuropsychological battery administered at JHU and used to derive four domain-specific cognitive composite scores, including executive function, episodic memory, language, and visuospatial processing (for details, see [29]). The average of the domain-specific scores served as a proxy for global cognition and was the primary cognitive outcome variable. Given our focus on the hippocampus, secondary analyses were restricted to the cognitive composite scores for episodic memory (Wechsler Memory Scale (WMS) logical memory delayed recall, WMS paired associates immediate recall, and California Verbal Learning Test total recall over trials 1 to 5) and visuospatial ability (Rey-Osterreith Complex Figure copy, Rey Figure Recall, and Wechsler Adult Intelligence Scale – Revised block design subtest) [24]. The JHU BIOCARD Clinical Core staff generates a consensus diagnosis annually using procedures comparable to those established by the National Institute on Aging (NIA) Alzheimer's Disease Centers program which have been published in detail in prior publications[30] and are described in greater details in the **Supplementary Material**.

### 2.2 MRI acquisition

MRI scans were conducted on a 3T Phillips Achieva scanner (Eindhoven, The Netherlands). The multi-modal imaging protocol encompassed a magnetization-prepared rapid gradient echo (MPRAGE) scan, which served as anatomical reference and for image registration purposes. These scans were acquired with the following parameters: TR = 6.7 ms, TE = 3.1 ms, shot interval of 3000 ms, flip angle of 8°, FOV = 240 × 256 mm², consisting of 170 slices with voxel dimensions of 1 × 1 × 1.2 mm³, and a scan duration of 5 min and 59 s. Diffusion-weighted images were acquired from a spin echo sequence (TR=7.5 s, TE=75 ms, FOV=260 × 260 mm², slice thickness=2.2 mm, flip angle=90°, b-value=700 s/mm^2^, number of gradients=33, 70 axial slices, 275 s scan duration).

### 2.3 Diffusion weighted image processing

The DWI images were pre-processed and segmented using MRICloud (https://braingps.mricloud.org; Mori et al., 2016), a fully automated pipeline. The tensor reconstruction and quality control followed the process implemented in DTIStudio (www.MRIStudio.org) [31]. Preprocessing included registration via a 9-mode affine transformation, simultaneously fitting both 6-mode rigid body motion and 3-mode gradient-dependent eddy-current distortions using normalized mutual information as the cost function. Subsequently, tensor reconstruction and quality control were performed in accordance with the DTIStudio pipeline [].

MRICloud applies a multi-atlas image parcellation algorithm that combines Large Deformation Diffeomorphic Metric Mapping (LDDMM; []) based on complementary contrasts (mean diffusivity (MD), fractional anisotropy (FA), and fiber orientation) together with a likelihood fusion method for DTI multi-atlas mapping and parcellation [32]. This procedure yielded 168 regions of interest (ROIs), from which the three eigenvalues of the diffusion tensor were extracted. Consistent with previous work [25,33], the analyses focused on MD values for the hippocampus, which has been shown to be reliably segmented with the fully automated MRICloud platform across a wide range of participant ages and levels of neurodegeneration [34]. For every voxel, MD was computed as the average of 3 primary eigenvalues of the diffusion tensor. MD values were extracted from ROI structural voxels that were identified as comprising of >50 % gray matter, and tensor metrics were further thresholded for this analysis to exclude potential white matter and cerebrospinal fluid partial volume contamination (fractional anisotropy < 0.2 and MD < 0.0015 mm^2^/s) [35,36]. To ensure that our choice of MD threshold (< 0.0015 mm²/s) did not arbitrarily affect our results, we conducted sensitivity analyses using additional thresholds (MD < 0.00133 mm²/s and MD < 0.00167 mm²/s). Hippocampal MD values remained essentially unchanged across these thresholds, with correlations exceeding 0.99 (*r* > 0.99, *p* < 0.001).

### 2.4 Volumetric image processing

Individual ROI volumes from the MPRAGE scans were also extracted from MRICloud. Hippocampal volumes were normalized to intracranial volume (HV/ICV × 100; unitless percent). Primary volumetric analyses used normalized hippocampal volume. In models evaluating hippocampal MD, normalized hippocampal volume was included as a covariate to mitigate potential atrophy-related CSF contamination in MD estimates. For transparency, raw hippocampal volume and ICV are reported alongside normalized values in Table 1. Results were substantively unchanged when using raw hippocampal volume with ICV entered as a covariate [35].

**Table 1:** Participant characteristics. Values reflect mean (SD) unless otherwise indicated.

Participant Characteristics	Total Sample (n = 190)	Cognitively Unimpaired (n = 176)	Mild Cognitive Impairment (n = 14)	p-value
Female sex, N ( %)	110 (58 %)	102 (58 %)	8 (57 %)	0.999
Age	67.6 (10.2)	66.9 (10.1)	76.9 (7.2)	< 0.001
Years of Education, years	17.3 (2.1)	17.4 (2.0)	16.4 (3.0)	0.332
Non-Hispanic White ( %)	164 (86 %)	151 (86 %)	13 (93 %)	0.697
APOE-ε4 Carriers, N ( %)	73 (39 %)	69 (39 %)	4 (29 %)	0.572
Hippocampal MD (mm/s^2^)	.0010 (<0.0001)	.0010 (<0.0001)	.0011 (<0.0001)	< 0.001
Hippocampal Volume (mm^3^)	6017 (626)	6017 (615)	6034 (780)	0.922
Intracranial Volume (mm^3^)	1510,803 (145,945)	1506,224 (140,147)	1568,374 (203,514)	0.475
Normalized Hippocampal Volume ( %)	0.40 (0.03)	0.40 (0.03)	0.39 (0.03)	0.093
Amyloid Positive Status ( %)	51 (27 %)	44 (25 %)	7 (50 %)	0.086
Braak I Tau SUVR	2.0 (0.9)	1.9 (0.8)	3.0 (1.4)	0.002
Braak II Tau SUVR	1.7 (0.6)	1.6 (0.4)	2.5 (1.3)	< 0.001
Braak III Tau SUVR	1.7 (0.5)	1.7 (0.4)	2.5 (1.2)	< 0.001
Cognitive Composite Score	−0.03 (0.7)	0.06 (0.6)	−1.16 (0.9)	<0.001
Episodic Memory Composite Score	0.01 (0.7)	0.08 (0.6)	−0.91 (0.7)	<0.001
Visuospatial Composite Score	−0.02 (0.5)	0.02 (0.5)	−0.57 (0.4)	<0.001

Notes: APOE = apolipoprotein E; MCI = Mild Cognitive Impairment; MD = Mean Diffusivity; SUVR = Standardized Uptake Value Ratio.

### 2.5 Amyloid and tau PET acquisition

PET imaging was performed on a GE DISCOVERY RX PET/CT scanner. Tau imaging used 18F-MK6240, with scans obtained 90 min post-injection. Amyloid imaging employed 11C-PiB, with scans acquired 40 min post-injection. Both tracers were administered intravenously, and PET images were reconstructed using the 3D OP-OSEM algorithm, corrected for attenuation, decay, and scatter. Standardized Uptake Values (SUV) were calculated and normalized to body weight and injected dose.

### 2.6 PET image analysis

Full details of the PET image analysis workflow have been published previously [12]. Briefly, PET images were spatially smoothed with a 6 mm Gaussian kernel, aligned to a mean volume using rigid-body registration, and averaged to create a mean PET image for each participant. This image was registered to the T1-weighted MRI using linear registration in FreeSurfer. Partial volume effects (PVE) were corrected using a geometric transfer matrix approach, based on segmented T1-weighted MRI. Standardized Uptake Value Ratios (SUVRs) were calculated using the pons for tau PET and cerebellar gray matter for amyloid PET.

### 2.7 Tau PET burden quantification

Tau burden was assessed using ROIs based on Braak staging of neurofibrillary tangles [8,9]. High-resolution medial temporal lobe (MTL) segmentations were obtained using Advanced Segmentation of Hippocampal Subfields (ASHS) and aligned with the participant’s T1-weighted MRI, as previously described [12]. For each Braak stage, SUVR values were averaged across relevant ROIs to quantify tau burden. For Braak stage I, ROIs included the entorhinal cortex and Brodmann area 35, which corresponds closely with the transentorhinal cortex [8,9,37]. Braak stage II encompasses Brodmann area 36 (entorhinal cortex), anterior hippocampus, and posterior hippocampus. For the later Braak stages III-VI, composite ROIs using the Desikan-Killiany atlas in FreeSurfer [38] were generated by averaging SUVR values across the multiple brain regions associated with each stage. Braak stage III included the amygdala, parahippocampal gyrus, fusiform gyrus, and lingual gyrus. In this analysis, we focused on tau burden in Braak stages I to III, as they include the hippocampus and adjacent structures. Most participants were cognitively unimpaired, and few would be expected to have significant tau burden in more advanced Braak stages which were therefore not considered in this study.

### 2.8 Amyloid burden quantification

Global amyloid burden was quantified by taking the averaging 11C-PiB uptake across several amyloid-associated regions, including the orbitofrontal cortex, precuneus, cingulate cortex, parietal cortex, temporal cortex, and superior frontal gyrus. A mean SUVR threshold of 1.40 was used to define amyloid positivity, consistent with prior studies [39].

### 2.9 APOE genotype

*APOE* genotyping was conducted by restriction enzyme digestion of PCR-amplified genomic DNA. Participants were classified dichotomously as ε4 carriers if they had at least one ε4 allele; otherwise, they were classified as non-carriers. *APOE* ε2/ε4 carriers were included with the ε4 carrier group, given evidence that their risk of AD pathology is more similar to that of ε3/ε4 than ε4 non-carriers [40].

### 2.10 Statistical analysis

Given the overall goal of examining whether hippocampal volume and microstructure moderate the association between tau burden in early Braak stages with cognition, we first evaluated whether higher tau burden in early Braak regions is indeed associated with worse cognitive performance. To do so, the first set of models used multivariate linear regression with tau PET signal in Braak stages I, II, or III as predictors and both global and domain-specific cognitive composite scores as outcome variables, using separate models for each Braak Stage and cognitive measure. The following covariates were included: age, sex, years of education, *APOE*-ε4 carrier status, amyloid status (PiB-PET negative vs. positive), diagnostic status (unimpaired vs. MCI), and time interval between DWI and tau PET scans.

Next, for those Braak stages where higher tau burden was significantly associated with lower cognitive scores, a second set of models tested whether hippocampal volume or hippocampal MD moderated the relationship between tau burden and cognitive performance. This was done by adding hippocampal measure (i.e., volume or MD) and the tau × hippocampal measure interaction terms to the regression models. If the tau x hippocampal measure interaction was significant, stratified analyses were conducted that divided participants into high and low hippocampal measure groups (based on a median split) and re-estimated the tau, cognition relationships within each subgroup. These models adjusted for the same covariates as the first set of models and for the time interval between the MRI and tau PET scans. Additionally, the models including hippocampal MD also covaried hippocampal volume, while the models including hippocampal volume similarly covaried hippocampal MD, allowing us to isolate the independent moderating effect of these variables.

To explore the clinical significance of hippocampal volume and microstructure in modifying the relationship between tau PET burden and cognition, a third set of models evaluated whether a) greater tau PET burden in Braak stages I, II, or III was associated with greater likelihood of having a diagnosis of MCI; and b) whether hippocampal volume or MD moderated the association between tau PET burden and diagnostic status (unimpaired vs. MCI). These analyses used binomial logistic regression models with diagnostic status as the outcome and included the same predictors and covariates as the second set of models (see above). Results are reported as standardized log-odds estimates, allowing for direct comparison with linear regression results.

### 2.11 Sensitivity analyses

To assess the robustness of our primary results, the first and second set of models were re-run in the subsample of participants classified as having elevated tau in Braak stages I, II, or III. The purpose of this analysis was to determine the pattern of results was the same among individuals with high tau burden in Braak regions I to III. Elevated tau was defined as having SUVR values >2 SDs above the mean for Braak stages I, II, or III compared to a reference group of cognitively unimpaired, amyloid-negative individuals under age 55 (reference group: *n* = 23; Braak I mean SUVR = 1.56, Braak II = 1.40, Braak III = 1.43), see [41] for similar approaches to defining elevated tau. The ‘elevated tau’ group consisted of all individuals classified as having elevated tau in Braak regions I, II or III. For this group, we then averaged tau burden across Braak I, II, and III and re-ran the first and second set of models.

Additional sensitivity analyses excluded participants diagnosed with MCI or "impaired, not MCI" to evaluate whether observed findings changed when symptomatic or cognitively impaired individuals were excluded. We also tested whether accounting for the time between amyloid-PET and tau-PET scans altered any of the primary findings.

All analyses were performed in R (v4.3.1). Regression models were checked for outliers and influential data points, flagged using the following criteria: leverage (hat value > 3 × mean), Cook’s distance > 0.5, or studentized residual > 3. Cases violating more than one criterion were excluded. Multicollinearity was assessed using variance inflation factors (VIF), with VIF > 5 considered indicative of concern. No data was excluded based on these exclusion criteria.

## 3 Results

Demographic and clinical characteristics of participants included in these analyses are shown in Table 1, separately for the full sample and by diagnostic status. Of the 190 participants, only 14 (7.4 %) were diagnosed with MCI. Those with MCI were significantly older, had lower cognitive scores, and showed significantly higher hippocampal MD. As expected, those with MCI had significantly greater tau-PET burden across all Braak stages (I–III). No group differences were observed in sex distribution, years of education, APOE-ε4 status, raw or normalized hippocampal volume, ICV, and amyloid positivity status.

### 3.1 Association of tau PET burden and cognition/diagnostic status

The first set of regression models showed that higher tau burden in Braak stages II and III was significantly associated with lower global cognitive performance (Braak II: β = −0.221, *p* = 0.007; Braak III: β = −0.217, *p* = 0.008), while Braak I tau level was not (β = −0.053, *p* = 0.518; see Table 2 for full model results). Similar patterns were observed for episodic memory, with tau burden in Braak II (β = −0.220, *p* = 0.009) and Braak III (β = −0.188, *p* = 0.024) negatively associated with episodic memory scores, but Braak I tau burden was not. Tau PET burden in Braak stages II and III was not associated with visuospatial performance (all *p* > 0.05), while greater Braak stage I tau burden was associated with better visuospatial abilities (β = 0.184, *p* = 0.037). In logistic regression models, higher tau burden in Braak stages II and III was associated with a greater likelihood of MCI diagnosis (Braak I: OR = 1.83 [0.97, 3.58], *p* = 0.063; Braak II: OR = 3.89 [1.53, 14.67], *p* = 0.013; Braak III: OR = 3.39 [1.37, 10.86], *p* = 0.015; see Table 2).

**Table 2:** Associations of Tau PET burden in braak stages I, II, and III with cognitive performance and diagnostic status.

Cognitive or Clinical Outcome Measure	Braak Stage Tau PET	Standardized Estimate	SE	p-value
				
Global Cognition	I	−0.053	0.082	0.518
	II	−0.221	0.082	0.007**
	III	−0.217	0.081	0.008**
				
Episodic Memory	I	−0.090	0.083	0.279
	II	−0.220	0.083	0.009**
	III	−0.188	0.083	0.024*
			
Visuospatial Abilities	I	0.184	0.088	0.037*
	II	0.105	0.090	0.243
	III	0.077	0.089	0.392
MCI Diagnostic Status (log-odds)	I	0.608	0.327	0.063
	II	1.359	0.548	0.013*
	III	1.220	0.502	0.015*

Note. Multiple linear regressions evaluated associations with cognitive scores and controlled for age, sex, days between diffusion and tau scan, education, diagnosis, PiB-PET positive status, APOE ε4 status, and normalized hippocampal volume. Binomial logistic regressions were used for estimating the log-odds ratio of MCI diagnostic status and controlled for age, sex, education, PiB-PET positive status, APOE ε4 status, hippocampal mean diffusion and hippocampal volume. Corresponding odds ratios were:.

Braak I: 1.83 [0.97, 3.58].

Braak II: 3.89 [1.53, 14.67].

Braak III: 3.39 [1.37, 10.86].

*p < 0.05; **p < 0.01; ***p < 0.001.

### 3.2 Interactions between tau PET burden and microstructure or hippocampal volume in relation to cognition

Given the negative associations between tau burden in Braak regions II and III with global cognition and episodic memory, we next examined if these relationships differed by hippocampal volume or MD. Hippocampal MD significantly moderated the association between tau PET burden in Braak stages II and III with both global cognition and episodic memory, as indicated by significant tau x hippocampal MD interactions (for global cognition: Braak II (β = −0.16, 95 % CI [−0.29, −0.04], *p* = 0.011) and Braak III (β = −0.18, 95 % CI [−0.30, −0.05], *p* = 0.005)); for episodic memory: Braak II (β = −0.15, 95 % CI [−0.28, −0.03], *p* = 0.019) and Braak III (β = −0.15, 95 % CI [−0.28, −0.02], *p* = 0.023; See Supplementary Table 1 for full model results).

**Fig. 1:**
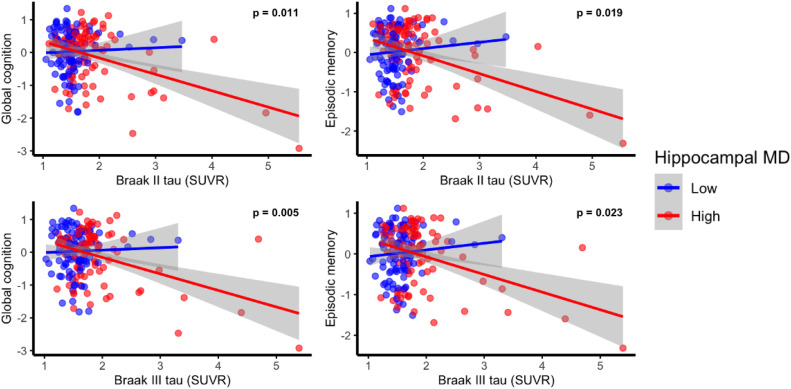
Lower hippocampal mean diffusion (MD) (i.e., better microstructure) attenuates the negative associations between tau PET burden Standardized Uptake Value Ratio (SUVR) in Braak stages II and III for global cognitive and episodic memory performance. Hippocampal MD levels are based on a median split while p-value is based on hippocampal MD as a continuous moderator with full covariate adjustment (age, sex, days between diffusion and tau scan, education, diagnosis, PiB-PET positive status, APOE ε4 status, and normalized hippocampal volume).

Models stratified by hippocampal MD (median split) demonstrated that among individuals with lower hippocampal MD (i.e., better microstructural integrity), tau burden in Braak II and III was not significantly associated with global cognition or episodic memory (all ps > 0.17). In contrast, among individuals with higher hippocampal MD (i.e., lower microstructural integrity), greater tau burden in Braak II and III was strongly associated with lower global cognitive performance (Braak II: β = −0.25, *p* = 0.009; Braak III: β = −0.25, *p* = 0.009) and episodic memory (Braak II: β = −0.26, *p* = 0.011; Braak III: β = −0.22, *p* = 0.032; **Supplementary Table** 2 and Fig. 1).

Normalized hippocampal volume did not significantly moderate the association between tau burden and global cognition or episodic memory for Braak stage II or III (all *ps* > 0.500),.

### 3.3 Moderating effect of hippocampal MD on the tau–diagnosis relationship across braak stages

In an exploratory analysis, hippocampal MD again significantly moderated the relationship between tau PET burden in Braak Stages II (β = 1.21, 95 % CI [0.23, 3.36], OR = 3.36, 95 %CI [1.26, 11.4], *p* = 0.033) and III (β = 1.44, 95 % CI [0.16, 2.49], OR = 3.63, 95 %CI[1.31, 13.3], *p* = 0.028) with likelihood of MCI diagnosis.

Stratified analyses indicated that among individuals with low hippocampal MD (better microstructural integrity), tau PET burden in Braak II and III was not significantly associated with MCI diagnosis. In contrast, for individuals with high hippocampal MD, greater tau burden in these regions was strongly associated with an increased likelihood of MCI (see **Supplementary Table 1 and 2**). No significant interaction was observed between hippocampal MD and tau burden in Braak Stage I with respect to diagnostic status (β = 0.70, 95 % CI [−0.02, 1.54], *p* = 0.070).

### 3.4 Sensitivity analyses

Accounting for the time between amyloid and tau-PET did not change any of the primary findings (data not shown). Excluding participants with a diagnosis of impaired not MCI (*n* = 37) for the full sample did not alter the direction or significance of any primary findings (data not shown). However, the relationship between tau PET burden with global or episodic memory was no longer significant when excluding the MCI participants (*n* = 14) (all *p*s > 0.300), nor were interactions between hippocampal MD and tau PET burden in relation to any cognitive measures (all ps > 0.500).

**Fig. 2:**
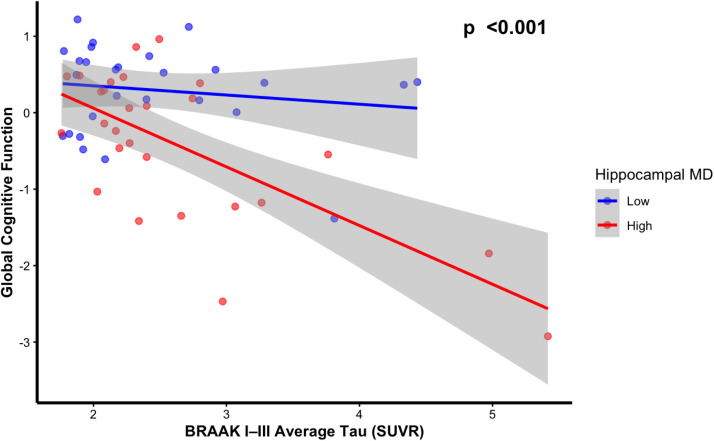
Hippocampal MD moderates the association between mean tau PET burden SUVR in Braak Stages I, II, and III with global cognition in a subsample of 54 participants with elevated tau PET burden in Braak regions I, II, or III. Hippocampal MD levels are based on a median split while p-value based on hippocampal MD as a continuous moderator with full covariate adjustment (age, sex, days between diffusion and tau scan, education, diagnosis, PiB-PET positive status, APOE ε4 status, and normalized hippocampal volume).

In the subset of 54 individuals classified as having elevated tau PET levels in Braak stages I, II, or III, hippocampal MD attenuated the negative association between average Braak I–III tau burden and global cognition (β = −0.310, 95 % CI [−0.48, −0.14], *p* < 0.001; see Fig. 2 a**nd Supplementary Table 3**). Tau PET was not significantly associated with global cognition among individuals with lower MD (*p* = 0490), whereas a significant negative association was observed among those with higher MD (*p* = 0.19; see **Supplementary Table 4**). A similar moderating effect of hippocampal MD was observed for episodic memory (β = −0.292, 95 % CI [−0.47, −0.12], *p* = 0.002). Finally, the moderating effect of hippocampal MD on the relationship between average Braak I–III tau burden and odds of MCI diagnosis was attenuated, although the effect did not reach statistical significance (β = 6.33, 95 % CI [1.67, 17.27], *p* = 0.073). However, when excluding the MCI participants (*n* = 9) the interactions between hippocampal MD and tau PET burden in relation to global cognition and episodic memory were no longer significant (see **Supplementary Table 5**).

## 4 Discussion

This study provides novel evidence that better hippocampal microstructure, indexed by lower DWI-derived MD, attenuates the negative relationship between tau PET burden and both cognitive performance and MCI status in dementia-free older adults. Specifically, individuals with lower hippocampal MD, reflecting better microstructural integrity, showed weaker associations between tau burden in Braak II and III regions with global cognition, episodic memory, and likelihood of MCI diagnosis. These associations remained significant in participants with elevated tau PET burden, highlighting the robustness of these findings. In contrast, hippocampal volume did not moderate the associations between tau burden and cognition. Taken together, these findings suggest that hippocampal microstructure, as indexed by MD, may be a potential biological mechanism underlying cognitive resilience to tau pathology in the hippocampus and adjacent structures. Our findings furthermore suggest that microstructural measures may be more sensitive markers of cognitive resilience to tau burden than volumetric measures, at least among dementia-free participants.

These findings contribute to a growing literature emphasizing that the clinical expression of AD pathology varies widely across individuals [5,13]. While tau PET burden is a well-established predictor of cognitive impairment and decline in older adults [2,10,12], our results suggest that preserved hippocampal microstructure may buffer against these effects, allowing some individuals to maintain higher than expected cognitive function despite brain pathology. This aligns with earlier studies demonstrating that hippocampal microstructure, more so than hippocampal volume, correlates with cognitive performance across the AD spectrum [[20], [21], [22], [23],^33^], and supports the relevance of hippocampal microstructure as a potential marker for cognitive resilience.

The moderating role of hippocampal MD was particularly evident for episodic memory, consistent with the hippocampus’s central role in episodic memory encoding and consolidation [24]. It is plausible that microstructural integrity of other brain regions outside of the MTL similarly supports resilience to tau pathology in other cognitive domains. In the current study we were not able to evaluate this possibility because our sample was composed primarily of cognitively unimpaired individuals, with limited tau pathology in regions outside of the MTL. However, in support of the view that microstructural integrity of regions other than the hippocampus supports resilience to tau pathology, a post-hoc exploratory analysis of the amygdala among participants with elevated tau PET in Braak regions I, II, or III showed that higher MD in the amygdala attenuated the negative association between tau PET burden and cognition (Supplementary Figure 1). Future studies are needed to confirm these findings and further evaluate the generality of microstructural integrity as a marker for cognitive resilience.

Although the neurobiological processes underlying DWI-derived hippocampal MD are not entirely clear, there are several mechanisms that could explain why hippocampal MD may provide cognitive resilience to tau pathology. First, current evidence suggests that gray matter MD may at least partially reflect synaptic density and integrity. For instance, a recent study found that lower hippocampal MD correlated with higher synaptic vesicle glycoprotein 2A (SV2A) PET binding, which serves as a marker of synaptic density, particularly in cognitively impaired, amyloid-positive older adults [42]. A complementary finding [43] suggested that DWI-based measures of intracellular diffusion in the hippocampus may correspond to loss of synaptic and dendritic complexity in tau susceptible regions. Lastly, animal studies have linked lower hippocampal MD to increased neuroplasticity [44,45]. It is possible, therefore that individuals with low MD have better synaptic and/or dendritic integrity, enabling them to maintain cognitive performance despite accumulating tau pathology. This would be in line with findings that synaptic dysfunction and loss are pivotal drivers of cognitive impairment in AD [46,47].

A second pathway by which low hippocampal MD may confer cognitive resilience to tau pathology is through the modulation of neuroinflammatory processes. In amyloid-positive individuals across the AD clinical spectrum, better hippocampal microstructural integrity has been associated with reduced PET signal from [¹⁸F]THK5351, a tracer now recognized to reflect astrogliosis and neuroinflammation due to off-target binding to monoamine oxidase-B (MAO-B), particularly in amyloid-positive individuals[48]. This supports the hypothesis that preserved microstructural integrity may signal a less neuroinflammatory environment in response to underlying tau burden, thereby helping to maintain cognitive performance. Further support comes from animal studies in which higher hippocampal MD is associated with greater microglial density and increased neuroinflammatory activity [49,50], highlighting MD’s sensitivity to a blend of microstructural features, including synaptic density and gliosis, that may underlie resilience against AD pathology.

Notably, tau accumulation is thought to drive subsequent neurodegeneration, microstructural changes (including greater MD[51]), and reductions in SVA2 synaptic PET signal[52]. Thus, individuals exhibiting high tau PET, combined with low MD, may be less susceptible to the detrimental effects of tau on microstructural integrity, thus maintaining preserved synaptic and dendritic function and enabling cognitive resilience. In this framework, such individuals could be considered as displaying resistance to tau pathology or as having better brain maintenance [7,13]. Alternatively, it is possible that individuals with high tau PET burden and lower MD had even lower MD (and better synaptic integrity) prior to the onset of tau accumulation, allowing them to tolerate greater pathological burden before cognitive decline ensues [3,5,15]. Finally, individuals with high tau burden and preserved hippocampal MD may also be in a less advanced stage of AD, where tau is present but has not yet significantly altered microstructural features (either because of resistance to tau) or because tau has not been present long enough). Longitudinal studies tracking tau, microstructural integrity, CSF or blood biomarkers, and cognition over time are needed to differentiate between these alternatives.

Our results align with broader evidence that cognitive resilience is related to neuroimaging markers that reflect aspects of brain integrity, including gray and white-matter microstructural integrity as well as functional connectivity. For instance, Qiu et al. (2024) recently reported that greater white matter microstructural integrity buffered the impact of tau PET on episodic memory decline among cognitively unimpaired individuals at familial risk for AD [14]. Similarly, Adams et al. (2022) demonstrated that functional network integrity mitigated the effects of amyloid PET on episodic memory performance [55]. Together, these findings support the idea that both structural and functional brain integrity contribute to domain-specific resilience, especially within episodic memory systems where early AD-related effects often manifest.

The present results showed that hippocampal volume did not moderate the association between tau PET burden and cognitive performance, despite its frequent use as a potential measure for cognitive resilience in prior studies [7,15,16]. This finding is consistent with emerging evidence that global volumetric measures may not be sensitive markers for cognitive resilience to tau pathology in dementia-free populations [13,20,22,23], though future studies are needed to examine this issue in more advanced disease stages. While postmortem studies have shown that hippocampal volume explains a modest proportion of cognitive variability beyond traditional neuropathological indices [53], such volumetric atrophy is generally interpreted as reflecting macroscopic atrophy and neuronal loss, processes that may predominantly emerge later in the course of AD.

The current findings are relevant to clinical intervention and prevention strategies for cognitive decline. Specifically, lower hippocampal MD has been associated with several modifiable lifestyle factors related to dementia risk and cognitive decline, including greater physical activity [25], better sleep quality [25], and a higher level of education [26]. This suggests that hippocampal MD could be used as an outcome measure in intervention studies aimed at reducing cognitive decline via these modifiable lifestyle factors [15,54]. Additionally, DWI-derived hippocampal MD may serve as a sensitive, non-invasive biomarker for identifying individuals who are cognitively resilient despite elevated levels of early AD pathology. As diffusion imaging is a non-invasive and repeatable imaging approach that is already widely used in clinical and research settings, incorporating microstructural metrics could enhance early risk stratification and inform potential intervention strategies. Furthermore, these results support a shift toward incorporating brain microstructure[42,48] into mechanistic models of cognitive aging and AD progression, particularly in preclinical and at-risk populations.

Nonetheless, several limitations warrant consideration. First, the cross-sectional design limits our ability to draw causal inferences. Our sample was predominantly non-Hispanic white and highly educated, limiting generalizability. Second, while the sample was enriched for individuals at increased risk for AD, including a higher proportion of APOE-ε4 carriers and participants with significant amyloid PET burden, few participants had MCI, limiting our ability to examine diagnostic differences and later stages of disease. In our primary analyses, diagnostic status was included as a covariate in all models. However, when the MCI subgroup was excluded, hippocampal MD did not significantly moderate the association between tau PET burden and cognition. This likely reflects the fact that in the cognitively unimpaired participants, the level of tau PET burden in Braak Stages II and III was too low to significantly impact cognitive performance, reducing our power to detect an interaction with hippocampal MD. These findings support the view that elevated tau PET burden outside of Braak Stage I represents a relatively late event during the preclinical phase of AD that is closely tied to the onset of cognitive impairment [1]. Furthermore, while the sample size for the primary analysis (*n* = 190) was moderate, the high-tau subgroup analysis was based on a small sample size (*n* = 54) and findings need to be confirmed in larger samples. Another limitation is the use of a single-shell diffusion sequence and tensor modeling to quantify hippocampal microstructure. Single-shell diffusion imaging restricts our use of advanced diffusion modeling techniques, such as multi-shell acquisition or neurite orientation dispersion and density imaging (NODDI), which provide enhanced specificity and improved correction for free water and CSF contamination. While our DTI processing steps were designed to mitigate atrophy-related bias and partial volume contamination, our findings should be interpreted as reflecting sensitivity to general microstructural alterations in the hippocampus rather than any specific neurophysiological process [25]. Future studies incorporating complementary measures, such as neuroinflammation, synaptic density, or functional activity, are needed to clarify the mechanisms underlying the observed associations. Ultimately, longitudinal approaches that integrate these advanced biomarkers will be essential to elucidate the temporal sequence and causal pathways linking hippocampal microstructure to resilience in the face of tau pathology in AD.

## Data availability statement

Data used in these analyses are available through standard application procedures described on the BIOCARD website (http://www.biocard-se.org). Additional information or materials relating to the analysis are available from the corresponding author (DC) upon reasonable request.

## Funding

This work was supported by the National Institutes of Health [grant numbers U19-AG033655, P30-AG005146, and 1K01AG092954)].

## CRediT authorship contribution statement

**Daniel D. Callow:** Writing – review & editing, Writing – original draft, Visualization, Methodology, Investigation, Formal analysis, Data curation, Conceptualization. **Nisha Rani:** Methodology, Data curation. **Kylie H. Alm:** Methodology, Data curation. **Corinne Pettigrew:** Writing – review & editing, Project administration, Methodology, Data curation. **Michael Miller:** Methodology. **Marilyn Albert:** Writing – review & editing, Supervision, Resources, Project administration, Methodology, Investigation, Funding acquisition, Formal analysis, Data curation, Conceptualization. **Arnold Bakker:** Writing – review & editing, Supervision, Methodology, Investigation, Data curation, Conceptualization. **Anja Soldan:** Writing – review & editing, Writing – original draft, Visualization, Supervision, Methodology, Investigation, Formal analysis, Data curation, Conceptualization.

## Declaration of competing interest

On behalf of all authors, the corresponding author states that there is no conflict of interest.

## Appendix Supplementary materials

Image, application 1
