# Supplementary material for: Hippocampal microstructure as a measure of cognitive resilience to tau PET burden in older adults
Source: J Prev Alzheimers Dis. 2026 Jan 6;13(2):100454. doi: 10.1016/j.tjpad.2025.100454 (PMC12869048; doi:10.1016/j.tjpad.2025.100454)
Supplement: Supplementary file 1 [file mmc1.docx]

Supplementary Materials for Manuscript Titled

“Hippocampal Microstructure as a Measure of Cognitive Resilience to Tau PET Burden in Older Adults”

Daniel D. Callow ^a^ Nisha Rani ^a^, Kylie H Alm^a^, Corinne Pettigrew^b^, Michael Miller^c^, Marilyn Albert^b^, Arnold Bakker^a,b^, Anja Soldan^b^, and the BIOCARD Research Team

^a^ Department of Psychiatry and Behavioral Sciences, Johns Hopkins University School of Medicine, Baltimore, MD

^b^ Department of Neurology, Johns Hopkins School of Medicine, Baltimore, MD

^c^ Department of Biomedical Engineering, Johns Hopkins University, Baltimore, MD

**Clinical Assessment**

The JHU BIOCARD Clinical Core staff generates a consensus diagnosis annually using procedures comparable to those established by the National Institute on Aging (NIA) Alzheimer's Disease Centers program. First, a syndromic diagnosis is generated based on the following data: (1) clinical data describing the medical, neurological, and psychiatric status of the individual; (2) reports of cognitive changes by the individual and collateral sources; and (3) decline in cognitive performance over time, based on the review of longitudinal data (and relative to age-matched published norms). Syndromic diagnostic categories included: (1) cognitively unimpaired, (2) MCI, (3) impaired not MCI, and (4) dementia. When a participant is believed to be cognitively impaired, a decision regarding the probable etiology of the syndrome is determined based on the clinical information provided at each visit, including the individual's medical history. Multiple etiologies for a single participant are possible. The consensus diagnostic protocol was guided by the recommendations outlined in the NIA/Alzheimer's Association working group reports for a diagnosis of MCI ^1^ and AD dementia ^2^. An "Impaired not MCI" diagnosis is given in the case of contrasting information from the CDR interview and cognitive test scores (i.e., participants or collateral source reported concerns for cognitive changes in daily life, but the cognitive testing did not indicate declines, or vice versa). Since participants with a diagnosis of "Impaired not MCI" do not meet MCI criteria, they were included with the group of unimpaired participants, consistent with prior publications (see ^3^).

1. Albert M, DeKosky ST, Dickson D, et al. The diagnosis of mild cognitive impairment due to Alzheimer’s disease: Recommendations from the National Institute on Aging-Alzheimer’s Association workgroups on diagnostic guidelines for Alzheimer’s disease. *Alzheimer’s and Dementia*. 2011;7(3):270-279. doi:10.1016/j.jalz.2011.03.008

2. McKhann GM, Knopman DS, Chertkow H, et al. The diagnosis of dementia due to Alzheimer’s disease: recommendations from the National Institute on Aging-Alzheimer’s Association workgroups on diagnostic guidelines for Alzheimer’s disease. *Alzheimers Dement*. 2011;7(3):263-269. doi:10.1016/J.JALZ.2011.03.005

3. Albert M, Soldan A, Gottesman R, et al. Cognitive changes preceding clinical symptom onset of mild cognitive impairment and relationship to ApoE genotype. *Curr Alzheimer Res*. 2014;11(8):773. doi:10.2174/156720501108140910121920

**Tables**

Supplementary Table 1. Full Model Outputs of Hippocampal MD Moderating Associations Between Mean Tau PET Burden and Cognition in Full Sample

| **Cognitive Measure** | **Term** | **Standardized Beta Estimate** | **SE** | **p-value** |
| --- | --- | --- | --- | --- |
|  |  |  |  |  |
| Global Cognition | Education | 0.095 | 0.064 | 0.141 |
|  | APOE-e4 Status | -0.013 | 0.138 | 0.926 |
|  | AGE | 0.057 | 0.100 | 0.564 |
|  | SEX | 0.544 | 0.130 | <.001*** |
|  | Tau Diffusion Days | 0.000 | 0.000 | 0.123 |
|  | Amyloid Status | -0.065 | 0.171 | 0.703 |
|  | Diagnosis | 1.222 | 0.288 | <.001*** |
|  | Tau Braak II | -0.054 | 0.103 | 0.601 |
|  | Hippocampal MD | 0.034 | 0.094 | 0.716 |
|  | Normalized Hippocampal Volume | 0.050 | 0.065 | 0.441 |
|  | Tau Braak II × Hippocampal MD | -0.164 | 0.064 | 0.011* |
|  |  |  |  |  |
| Global Cognition | Education | 0.097 | 0.064 | 0.133 |
|  | APOE-e4 Status | -0.013 | 0.138 | 0.923 |
|  | AGE | 0.053 | 0.099 | 0.595 |
|  | SEX | 0.538 | 0.130 | <.001*** |
|  | Tau Diffusion Days | 0.000 | 0.000 | 0.113 |
|  | Amyloid Status | -0.065 | 0.171 | 0.705 |
|  | Diagnosis | 1.220 | 0.285 | <.001*** |
|  | Tau Braak III | -0.046 | 0.100 | 0.645 |
|  | Hippocampal MD | 0.033 | 0.094 | 0.722 |
|  | Normalized Hippocampal Volume | 0.053 | 0.065 | 0.416 |
|  | Tau Braak III × Hippocampal MD | -0.179 | 0.063 | 0.005** |
|  |  |  |  |  |
| Episodic Memory | Education | 0.043 | 0.065 | 0.511 |
|  | APOE-e4 Status | 0.070 | 0.140 | 0.615 |
|  | AGE | 0.130 | 0.101 | 0.201 |
|  | SEX | 0.503 | 0.132 | <.001*** |
|  | Tau Diffusion Days | 0.000 | 0.000 | 0.050 |
|  | Amyloid Status | -0.055 | 0.174 | 0.751 |
|  | Diagnosis | 1.214 | 0.293 | <.001*** |
|  | Tau Braak II | -0.064 | 0.105 | 0.540 |
|  | Hippocampal MD | 0.061 | 0.096 | 0.526 |
|  | Normalized Hippocampal Volume | 0.086 | 0.066 | 0.197 |
|  | Tau Braak II × Hippocampal MD | -0.153 | 0.065 | 0.019* |
|  |  |  |  |  |
| Episodic Memory | Education | 0.043 | 0.066 | 0.509 |
|  | APOE-e4 Status | 0.070 | 0.141 | 0.618 |
|  | AGE | 0.132 | 0.102 | 0.196 |
|  | SEX | 0.490 | 0.133 | <.001*** |
|  | Tau Diffusion Days | 0.000 | 0.000 | 0.048 |
|  | Amyloid Status | -0.075 | 0.176 | 0.672 |
|  | Diagnosis | 1.268 | 0.293 | <.001*** |
|  | Tau Braak III | -0.047 | 0.102 | 0.646 |
|  | Hippocampal MD | 0.055 | 0.096 | 0.570 |
|  | Normalized Hippocampal Volume | 0.089 | 0.067 | 0.187 |
|  | Tau Braak III × Hippocampal MD | -0.148 | 0.065 | 0.023* |
|  |  |  |  |  |
| Diagnostic Status (log-odds) | Education | -0.657 | 0.325 | 0.043* |
|  | APOE-e4 Status | -0.051 | 0.967 | 0.958 |
|  | AGE | 0.057 | 0.709 | 0.935 |
|  | SEX | 0.210 | 0.786 | 0.789 |
|  | Tau Diffusion Days | 0.002 | 0.001 | 0.035* |
|  | Amyloid Status | -0.280 | 1.140 | 0.806 |
|  | Tau Braak II | -0.221 | 0.671 | 0.742 |
|  | Hippocampal MD | 1.145 | 0.556 | 0.040 |
|  | Normalized Hippocampal Volume | -0.474 | 0.447 | 0.289 |
|  | Tau Braak II × Hippocampal MD | 1.214 | 0.571 | 0.033* |
|  |  |  |  |  |
| Diagnostic Status (log-odds) | Education | -0.694 | 0.324 | 0.032* |
|  | APOE-e4 Status | -0.089 | 0.971 | 0.927 |
|  | AGE | 0.096 | 0.708 | 0.892 |
|  | SEX | 0.292 | 0.788 | 0.711 |
|  | Tau Diffusion Days | 0.002 | 0.001 | 0.031* |
|  | Amyloid Status | -0.182 | 1.104 | 0.869 |
|  | Tau Braak III | -0.363 | 0.560 | 0.516 |
|  | Hippocampal MD | 1.187 | 0.556 | 0.033* |
|  | Normalized Hippocampal Volume | -0.565 | 0.450 | 0.209 |
|  | Tau Braak III × Hippocampal MD | 1.440 | 0.656 | 0.028* |
|  |  |  |  |  |
| **Note: Multiple linear and logistic regressions looking at moderating role of hippocampal microstructure on relationship between Tau PET Braak II and III burden with cognition and clinical status controlling for age, sex, days between diffusion and tau scan, education, diagnosis, PiB-PET status, APOE ε4 status, and normalized hippocampal volume. *p < 0.05; **p < 0.01; ***p < 0.001* | | | | |

Supplementary Table 2. Braak Stage Tau PET and Cognition Relationships Stratified by Low and High Hippocampal Mean Diffusivity in Full Sample

| **Cognitive Measure** | **Braak Stage Tau PET** | **MD Group** | **Standardized Estimate** | **SE** | **p-value** |
| --- | --- | --- | --- | --- | --- |
|  |  |  |  |  |  |
| Global Cognition | II | Low | 0.069 | 0.19 | 0.718 |
|  | II | High | -0.251 | 0.09 | 0.009** |
|  | III | Low | 0.068 | 0.19 | 0.720 |
|  | III | High | -0.247 | 0.09 | 0.009 |
| Episodic Memory | II | Low | 0.06 | 0.19 | 0.749 |
|  | II | High | -0.259 | 0.10 | 0.011* |
|  | III | Low | 0.068 | 0.19 | 0.717 |
|  | III | High | -0.216 | 0.10 | 0.032* |
| MCI Diagnosis (log-odds) | II | Low | 28.86 | 69021.56 | 0.999 |
|  | II | High | 1.27 | 0.53 | 0.016* |
|  | III | Low | 36.04 | 31228.12 | 0.999 |
|  | III | High | 1.05 | 0.41 | 0.011* |
| **Note: Stratified (based on median split of hippocampal mean diffusivity) multiple linear regressions controlling for age, sex, education, diagnosis, PiB-PET positive status, APOE ε4 status, and hippocampal volume. Binomial logistic regression for Mild Cognitive Impairment (MCI) diagnostic status controlling for age, sex, education, PiB-PET positive status, APOE ε4 status, hippocampal mean diffusion, and hippocampal volume. We note that estimates and standard errors for the Low MD Group for estimating MCI diagnosis are so large due to their only being a small number (n=2) of MCI subjects in this group. *p-value < .05; **p-value < .01; ***p-value < .001.* | | | | | |

Supplementary Table 3. Full Model Outputs of Hippocampal MD Moderating Associations Between Mean Tau PET Burden and Cognition in Individuals with Elevated Tau PET

| **Cognitive Measure** | **Term** | **Standardized Beta Estimate** | **SE** | **p-value** |
| --- | --- | --- | --- | --- |
|  |  |  |  |  |
| Global Cognition | Education | -0.062 | 0.091 | 0.498 |
|  | APOE-e4 Status | -0.458 | 0.195 | 0.024* |
|  | AGE | 0.116 | 0.106 | 0.278 |
|  | SEX | 0.750 | 0.202 | <.001*** |
|  | Tau Diffusion Days | 0.151 | 0.097 | 0.128 |
|  | Amyloid Status | -0.201 | 0.201 | 0.322 |
|  | Diagnosis | 0.782 | 0.314 | 0.017* |
|  | Tau Braak I–III | -0.307 | 0.111 | 0.008** |
|  | Hippocampal MD | -0.129 | 0.123 | 0.297 |
|  | Normalized Hippocampal Volume | 0.087 | 0.098 | 0.382 |
|  | Tau Braak I–III × Hippocampal MD | -0.310 | 0.086 | <.001*** |
|  |  |  |  |  |
| Episodic Memory | Education | -0.109 | 0.091 | 0.237 |
|  | APOE-e4 Status | -0.377 | 0.196 | 0.061 |
|  | AGE | 0.188 | 0.106 | 0.083 |
|  | SEX | 0.678 | 0.203 | 0.002** |
|  | Tau Diffusion Days | 0.238 | 0.098 | 0.019* |
|  | Amyloid Status | -0.188 | 0.201 | 0.357 |
|  | Diagnosis | 0.663 | 0.315 | 0.041* |
|  | Tau Braak I–III | -0.329 | 0.112 | 0.005** |
|  | Hippocampal MD | -0.183 | 0.123 | 0.145 |
|  | Normalized Hippocampal Volume | 0.173 | 0.098 | 0.086 |
|  | Tau Braak I–III × Hippocampal MD | -0.292 | 0.086 | 0.002** |
|  |  |  |  |  |
| Diagnostic Status (log-odds) | Education | -0.974 | 0.734 | 0.185 |
|  | APOE-e4 Status | 0.530 | 3.100 | 0.864 |
|  | AGE | -1.118 | 2.024 | 0.581 |
|  | SEX | -2.777 | 3.459 | 0.422 |
|  | Tau Diffusion Days | -0.006 | 0.010 | 0.573 |
|  | Amyloid Status | -0.772 | 3.218 | 0.810 |
|  | Tau Braak I–III | 3.774 | 3.369 | 0.263 |
|  | Hippocampal MD | 3.249 | 2.395 | 0.175 |
|  | Normalized Hippocampal Volume | 2.561 | 2.101 | 0.223 |
|  | Tau Braak I–III × Hippocampal MD | 6.327 | 3.535 | 0.073 |
|  |  |  |  |  |
| **Note: Tau PET burden was averaged across Braak stages I–III among individuals with elevated tau PET. Multiple linear and logistic regressions examined the moderating role of hippocampal microstructure on the relationship between tau PET burden, cognition, and clinical status, controlling for age, sex, days between diffusion and tau scan, education, diagnosis, PiB-PET status, APOE ε4 status, and normalized hippocampal volume.*  **p < 0.05; **p < 0.01; ***p < 0.001.* | | | | |

Supplementary Table 4. Association between Mean Tau PET Burden and Cognition, Stratified by Low and High Hippocampal Mean Diffusivity in Individuals with Elevated Tau PET

| **Cognitive Measure** | **MD Group** | **Standardized Estimate** | **SE** | **p-value** |
| --- | --- | --- | --- | --- |
|  |  |  |  |  |
| Global Cognition | Low | -0.102 | 0.14 | 0.490 |
|  | High | -0.549 | 0.21 | 0.019* |
|  |  |  |  |  |
| Episodic Memory | Low | -0.168 | 0.15 | 0.273 |
|  | High | -0.608 | 0.24 | 0.012* |
|  |  |  |  |  |
| **Note: Tau PET burden was averaged across Braak stages I, II, and III among individuals classified as having elevated tau PET in Braak I, II, or III. Results are from stratified (based on median split of hippocampal mean diffusivity) multiple linear regressions controlling for age, sex, education, diagnosis, PiB-PET positive status, APOE ε4 status, and hippocampal volume.*p-value < .05; **p-value < .01; ***p-value < .001.* | | | | |

Supplementary Table 5. Full Model Outputs of Hippocampal MD Moderating Associations Between Mean Tau PET Burden and Cognition in Individuals with Elevated Tau PET excluding MCI individuals (n=46)

| **Cognitive Measure** | **Term** | **Standardized Beta Estimate** | **SE** | **p-value** |
| --- | --- | --- | --- | --- |
|  |  |  |  |  |
| Global Cognition | Education | -0.0316 | 0.14 | 0.826 |
|  | APOE-e4 Status | -0.732 | 0.32 | 0.027* |
|  | AGE | 0.163 | 0.16 | 0.315 |
|  | SEX | 1.069 | 0.31 | 0.002** |
|  | Tau Diffusion Days | 0.305 | 0.15 | 0.055 |
|  | Amyloid Status | -0.200 | 0.30 | 0.506 |
|  | Tau Braak I–III | -0.282 | 0.17 | 0.108 |
|  | Hippocampal MD | 0.015 | 0.19 | 0.938 |
|  | Normalized Hippocampal Volume | 0.082 | 0.16 | 0.613 |
|  | Tau Braak I–III × Hippocampal MD | -0.225 | 0.23 | 0.334 |
|  |  |  |  |  |
| Episodic Memory | Education | -0.117 | 0.130 | 0.376 |
|  | APOE-e4 Status | -0.580 | 0.291 | 0.054 |
|  | AGE | 0.182 | 0.147 | 0.224 |
|  | SEX | 1.052 | 0.287 | 0.001** |
|  | Tau Diffusion Days | 0.378 | 0.141 | 0.012* |
|  | Amyloid Status | -0.192 | 0.272 | 0.484 |
|  | Tau Braak I–III | -0.353 | 0.157 | 0.031* |
|  | Hippocampal MD | -0.043 | 0.174 | 0.805 |
|  | Normalized Hippocampal Volume | 0.134 | 0.148 | 0.371 |
|  | Tau Braak I–III × Hippocampal MD | -0.293 | 0.210 | 0.172 |
| **Note: Tau PET burden was averaged across Braak stages I–III among individuals with elevated tau PET. Multiple linear and logistic regressions examined the moderating role of hippocampal microstructure on the relationship between tau PET burden, cognition, and clinical status, controlling for age, sex, days between diffusion and tau scan, education, diagnosis, PiB-PET status, APOE ε4 status, and normalized hippocampal volume.*  **p < 0.05; **p < 0.01; ***p < 0.001.* | | | | |

**Figures**


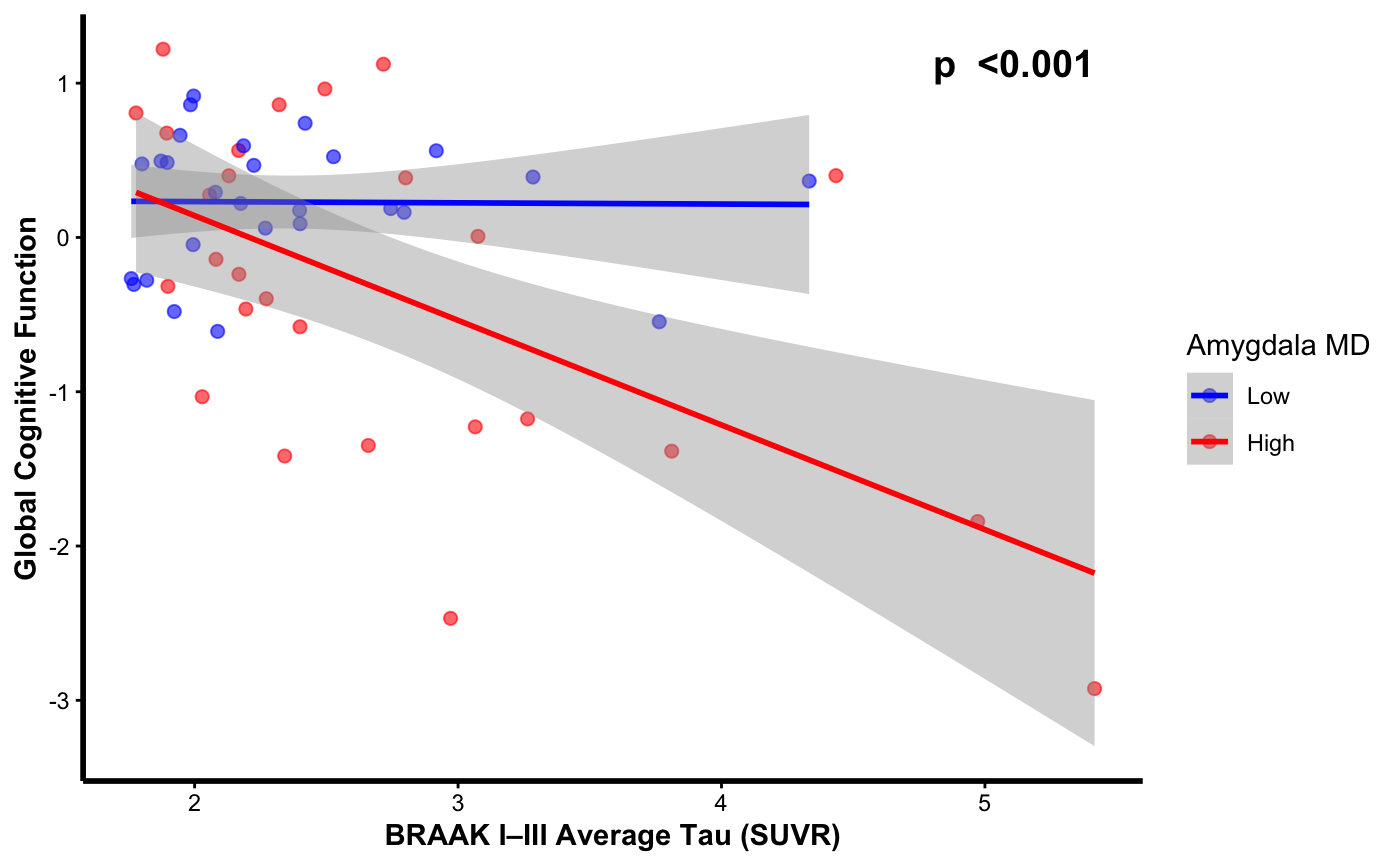


Supplementary Figure 1: Exploratory analysis of amygdala MD moderating the association between mean tau PET burden (SUVR) in Braak Stages I, II, and III and global cognition in a subsample of 54 participants with elevated tau PET burden. These findings suggest that gray matter microstructure may be sensitive to underlying mechanisms that promote cognitive resilience to tau burden. The amygdala MD was selected for this exploratory analysis due to its proximity to the hippocampus within the medial temporal lobe (MTL), where significant tau burden was observed in our sample, and because it can generally be segmented accurately. We did not observe that amygdala MD moderated any tau-cognition relationships in the full sample, potentially due to low tau burden in this larger cohort. Caution is warranted in interpreting these findings due to the amygdala's smaller size compared to the hippocampus, which increases the likelihood of partial volume effects in diffusion measurements, as well as the limited prior research on amygdala MD in relation to aging, memory, and Alzheimer's disease.
